# Assisted exploration of difficult transitions in humanoid motion tracking

**DOI:** 10.3389/fnbot.2026.1920711

**Published:** 2026-09-30

**Authors:** Chaojie Fu, Chengkai Su, Lei Jiang, Chengwei Zhuo, Kaixin Lan, Shaowen Cheng, Hongtao Wang

**Affiliations:** 1 The Center for X-Mechanics, Zhejiang University, Hangzhou, Zhejiang, China; 2 Department of Engineering Mechanics, The Institute of Applied Mechanics, Zhejiang University, Hangzhou, Zhejiang, China; 3 Zhejiang University (ZJU)-Hangzhou Global Scientific and Technological Innovation Center, Zhejiang University, Hangzhou, Zhejiang, China; 4 The State Key Laboratory of Fluid Power and Mechatronic System, Zhejiang University, Hangzhou, Zhejiang, China

**Keywords:** dynamic motion control, humanoid robot, motion tracking, reinforcement learning, robot learning, whole body control

## Abstract

Humanoid motion tracking provides a scalable interface for transferring kinematic human motion to whole-body robot control, but highly dynamic and contact-rich references remain difficult because underactuation and discontinuous contacts can restrict the closed-loop state distributions explored during training. External wrenches can reduce dynamic mismatch during imitation, but retaining such assistance in the final controller can compromise physical realism. This paper proposes residual-wrench-assisted reinforcement learning (RWARL), a two-stage framework that uses base residual wrenches as a temporary optimization scaffold. RWARL first solves a relaxed tracking problem with residual-wrench assistance and then anneals the residual-wrench strength while adapting early-termination bounds and segment sampling to observed training difficulty. To keep the policy adaptable as the training distribution shifts from assisted to unassisted dynamics, RWARL incorporates plasticity-preserving mechanisms during relaxation. Experiments on highly dynamic and contact-rich references show that RWARL generally improves global tracking accuracy, with the largest gains near difficult transitions where standard training tends to produce conservative behavior. Compared with the baseline, the final unassisted RWARL policies reduce global body-position tracking error by 8.6%–37.8% on the highly agile and contact-rich motions examined in this work and achieve the largest successful-frame-rate gain of 41.8 percentage points on *jump twist*. A closed-loop predecessor-state diagnostic further links these gains to more favorable predecessor-state distributions around critical phases. We deploy the final unassisted controllers on Unitree G1 hardware, demonstrating that the learned policies remain executable after residual assistance is removed. Together, these findings suggest that RWARL uses residual wrenches to reshape learning around difficult, low-reachability transitions, allowing the final unassisted policy to inherit better tracking behavior in phases that standard training tends to underexplore.

## 1 Introduction

Humanoid motion tracking has emerged as a central paradigm for whole-body humanoid robot control, enabling robots to reproduce diverse human motions ranging from locomotion to athletic and highly dynamic skills. A recent survey places physics-based imitation for humanoid robots within the broader landscape of imitation learning for legged locomotion (Mirza and Singh, 2025). The tracking problem studied here belongs to this physics-based imitation setting: a policy is trained to control a humanoid robot to track a reference motion derived from human motion data. Motion tracking provides a scalable way to transfer human motion data to humanoid robots, and the ability to accurately track reference motions is a key prerequisite for general-purpose humanoid control.

Early physics-based imitation systems and specialist humanoid trackers focused primarily on reproducing individual or narrowly defined motion clips (Peng et al., 2018; He et al., 2025; Xie et al., 2026; Liao et al., 2025). Recent methods instead train deployable generalist trackers that cover large motion sets with a single policy (Chen et al., 2025; Yin et al., 2025). Motion tracking is increasingly treated as a foundation task: single policies are trained over broad motion datasets, and the learned skills are reused as interfaces for downstream control, teleoperation, and multimodal control (Han et al., 2025; Luo et al., 2026; Sun et al., 2026). This shift improves motion coverage, but it also makes tracking a heterogeneous control problem where different motion types can impose conflicting control requirements (Wang et al., 2025). The difficulty becomes especially visible for diverse, highly dynamic, and contact-rich reference motions (Wang et al., 2026; Zhang et al., 2025), where small deviations from the target trajectory can lead to balance loss or complete failure. Work on highly dynamic tracking frames this scaling tension as a generality barrier, where tracking fidelity can degrade as motion diversity and physical executability demands increase (Wang et al., 2026). Related work on agility-stability control shows a similar tension from another angle, where balance-oriented and agility-oriented objectives can pull a unified policy in different directions (Pan et al., 2026). Therefore, the central question is not only whether a humanoid can complete a reference motion, but where and why tracking fidelity breaks down within the motion.

Existing methods often report aggregate tracking metrics over broad motion libraries, but such averages can obscure phase-specific failures or performance degradation. In practice, a tracking policy may appear accurate in terms of average error while still being inaccurate near contact transitions, aerial phases, sharp turns, or highly constrained balance configurations. Diagnostic benchmarks often separate motion types and difficulty levels because aggregate scores can mask methods that perform well only on a narrow subset of motions (Li et al., 2026a). Highly dynamic motion datasets further show that ordinary motion libraries may not stress the controller near failure boundaries such as rapid center-of-mass shifts, abrupt posture changes, and fall recovery (Lei et al., 2026).

Even when tracking failures are localized to specific motion phases, their underlying causes remain coupled rather than singular. Reference infeasibility can contribute to such failures when transferred human motions contain floating, ground penetration, or inconsistent center-of-mass behavior, thereby creating a conflict between reference tracking and physical stability (Li et al., 2026b). Limited exploration under the robot's own simulated dynamics can further restrict the state distribution on which tracking behavior is learned. Prior work shows that physics-based imitation depends strongly on rollout-state coverage through reference-state initialization and early termination (Peng et al., 2018), and that dynamic skills can hinge on critical states such as a backflip takeoff (Ma et al., 2021). In humanoid tracking, underactuation and discontinuous contacts can therefore make the predecessor states and contact sequence needed for accurate tracking difficult to discover even in simulation.

External assistance has long been used to expand the feasible control space for physics-based humanoid learning. In residual-force formulations, this assistance is retained as part of the final simulated policy, while methods differ in where the virtual input enters the dynamics. Prior work applies residual forces or wrenches to selected body links, root degrees of freedom, foot contacts, or the physics module used for pose estimation (Yuan and Kitani, 2020; Luo et al., 2022, 2021). These mechanisms can improve tracking coverage and success rates, but persistent external forces can also act as non-physical stabilization. PHC therefore removes residual forces from the controller to improve physical realism (Luo et al., 2023). This concern motivates treating assistance as a temporary training scaffold rather than as a permanent control input.

Recent scaffold-based methods instead use assistance during training while requiring the final policy to operate without support. They differ primarily in assistance target and annealing strategy. In terms of target, scaffolds may support the robot body or base (Huang et al., 2025), including A2CF (Cao et al., 2025), EFGCL (Yoneda et al., 2026), and ZEST (Sleiman et al., 2026); guide contact interactions (Pan et al., 2025); or assist manipulated objects during loco-manipulation (Zhao et al., 2025). In terms of annealing, assistance can be reduced through scheduled force decay or success-conditioned schedules (Huang et al., 2025), adaptive bounds and masking, sampling curricula, or gradual relaxation of contact guidance and object-assistance gains (Pan et al., 2025; Zhao et al., 2025). These choices matter because the assistance target changes the dynamics experienced by the policy, and the annealing strategy shapes how close the learned behavior becomes to the original unassisted control problem.

A closer comparison of the body- and base-assistance methods reveals different ways of producing the assisting wrench. A2CF trains a separate assistive policy that has access to additional state information and chooses a base wrench for the robot's current state. EFGCL instead relies on force patterns designed for each task, including where and when the forces are applied. ZEST calculates torso wrenches directly from a model of the robot dynamics and the current base-tracking error. Like A2CF, our method learns the assistance rather than prescribing it, but predicts small adjustments to the desired base pose, which the residual-wrench controller converts into forces and torques.

They also differ in how assistance is withdrawn as learning progresses. A2CF adjusts the allowable force range according to force use and skill progress, and occasionally removes assistance during training. EFGCL reduces the overall assistance level after the task success rate reaches a prescribed threshold. ZEST and our method both focus training on difficult motion segments, but use this information differently. ZEST sets the assistance level separately for each segment and uses the same difficulty estimate to choose where along the reference motion each training episode begins. Our method reduces a single overall assistance scale after performance has improved across all segments. For each segment, it separately adjusts how much tracking error is allowed before an episode ends and how often training begins from that segment, based on its failure frequency and the amount of residual assistance it requires.

The methods are also designed for different learning settings. A2CF emphasizes faster acquisition of walking, backflip, and dance skills; EFGCL addresses sparse-reward exploration for quadrupedal jumping and flipping without reference trajectories; and ZEST targets motion transfer across different reference sources and robot embodiments. Our method focuses on difficult transitions in reference-motion tracking and on maintaining policy adaptability as learned assistance is removed. The predecessor-state diagnostic then examines how the final unassisted policy approaches these transitions.

Even as a scaffold, assistance changes training dynamics by expanding the action space and compensating for reference-simulation mismatch (Yuan and Kitani, 2020; Luo et al., 2022). However, if assistance is too strong or retained too long, the policy may rely on an artificial support that will not exist at deployment (Luo et al., 2023). This motivates careful choices of assistance location, magnitude, and annealing schedule.

Annealing assistance changes the dynamics, state distribution, and gradient signals seen by the policy, raising a plasticity concern. Neural networks can lose adaptability under continual or non-stationary training (Dohare et al., 2024; Lyle et al., 2023), and warm-started models can generalize worse than models retrained from scratch (Ash and Adams, 2020). In on-policy RL, similar degradation has been observed under controlled distribution shifts with PPO, where warm-started policies can underperform reset policies and some mitigation methods transfer unreliably (Juliani and Ash, 2024). Thus, an assisted tracker may remain specialized to assisted rollouts unless relaxation keeps the policy trainable as support disappears.

To address these limitations, we propose residual-wrench-assisted RL (RWARL), a two-stage framework for highly dynamic and contact-rich humanoid motion tracking. RWARL first solves a relaxed tracking problem with temporary base residual wrenches, improving exploration around difficult motion phases and predecessor states. It then anneals the residual scale toward zero while continuing policy optimization. The final controller is evaluated without external assistance. During relaxation, RWARL adapts feasible bounds and segment sampling from observed failure rates and residual-wrench magnitudes, reducing dependence on fixed hand-designed curricula. It also uses plasticity-preserving mechanisms to maintain adaptability as the training dynamics shift from assisted to unassisted control.

The main contributions of this work are:

- We propose RWARL, a residual-relaxation training framework that combines temporary base-wrench assistance with adaptive relaxation and sampling, and show that it improves tracking accuracy on highly dynamic and contact-rich motions.
- We validate that policy plasticity loss is a practical issue in residual-relaxation training, where the policy must adapt under continuously shifting dynamics.
- We provide a predecessor-state reachability diagnostic showing that temporary residual assistance enables broader exploration around reference motions and helps the policy discover better predecessor states for difficult motion segments.
- We deploy the final unassisted policy on Unitree G1 hardware to validate the dynamic feasibility of the trained policy.

The structure of the paper is as follows. Section 2 presents the RWARL formulation and training procedure. Section 3 reports the simulation experiments, ablation studies, predecessor-state analysis, and hardware validation. Section 4 discusses the results, limitations, and future directions.

## 2 Method

In this section, we present residual-wrench-assisted reinforcement learning (RWARL), a two-stage training framework for highly agile and contact-rich humanoid motion tracking. The first stage solves a relaxed tracking problem in which an auxiliary residual wrench is applied to the robot base. These residual wrenches serve as temporary assistance, improving exploration and optimization on difficult motion segments. The second stage then recovers a physically feasible controller by progressively annealing residual assistance while continuing policy optimization under increasingly realistic dynamics.

### 2.1 Tracking problem and motivation

We formulate motion tracking as a Markov decision process (MDP), as described in Equation 1.


$$\begin{array}{l} M=\left(S,A,P,r,\gamma \right). \end{array}$$ (1)

At time *t*, ${\text{s}}_{t}\in S$ denotes the complete environment state, ${\text{a}}_{t}\in A$ denotes the action, *P*(**s**_*t*+1_∣**s**_*t*_, **a**_*t*_) denotes the transition probability, *r* denotes the reward function, and γ denotes the discount factor. Let **x**_*t*_ denote the component of **s**_*t*_ which contains robot states, and let **g**_*t*_ denote the corresponding reference robot state at the same frame. The observation ${\text{o}}_{t}=\Omega \left({\text{s}}_{t},{\text{g}}_{t}\right)\in O$ is constructed from the simulator state and reference information available to the policy, where Ω denotes the observation map. The policy π samples an action **a**_*t*_ ~ π(·∣**o**_*t*_), typically a set of joint-level control targets. The objective is to learn a policy whose induced robot state **x**_*t*_ remains close to the reference trajectory.

Despite recent progress in agile and large-scale humanoid tracking (Liao et al., 2025; Chen et al., 2025; Yin et al., 2025), standard RL training can still yield conservative controllers for highly dynamic or contact-rich motions, where policies preserve robustness by accepting larger tracking errors near contact transitions and constrained interactions (Wang et al., 2026; Zhang et al., 2025).

We view this behavior not only as a robustness-accuracy trade-off, but also as a consequence of insufficient exploration under complex humanoid dynamics. Even when tracking rewards are dense, underactuation and discontinuous contacts can make useful state transitions difficult to discover and optimize. Humanoid robots are underactuated at the floating base, and many agile motions rely on intermittent contacts with the environment. These constraints reduce controllability and make contact-rich trajectories difficult to track reliably.

RWARL addresses this difficulty by relaxing the tracking problem with auxiliary residual wrenches and then gradually removing the assistance. The residual wrenches are used only as a training scaffold. They improve exploration and optimization in difficult regions of the trajectory, while the relaxation stage transfers the learned behavior back to the original underactuated system. The training pipeline is illustrated in Figure 1.

**Figure 1:**
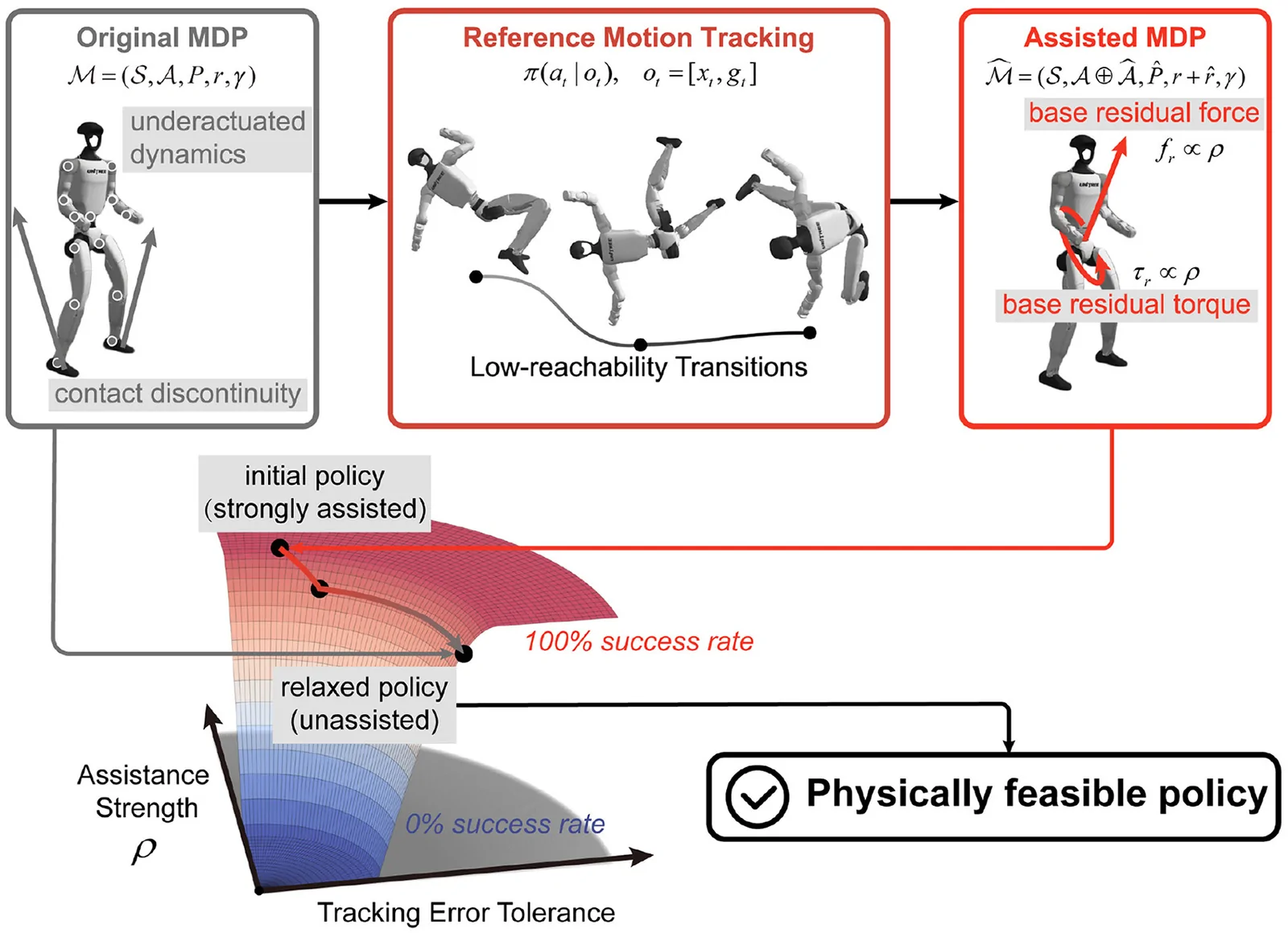
Overview of RWARL. Highly dynamic humanoid motion tracking in the original MDP is difficult because underactuation and contact discontinuities create low-reachability transitions. RWARL first trains in an assisted MDP, where base residual forces and torques scaled by ρ provide temporary assistance. It then anneals ρ toward a small training lower bound while continuing policy optimization, and the final deployable policy is evaluated without residual assistance. The bottom surface illustrates success rate over assistance strength and tracking-error tolerance: increasing ρ improves reachability under a fixed tolerance, while loosening the tolerance accepts larger tracking deviations under fixed assistance. The objective is to obtain the most accurate policy after residual assistance is disabled for evaluation, when the policy no longer depends on external assistance.

### 2.2 Residual-wrench-assisted tracking

We augment the original MDP with an additional residual action space $\hat{A}$. For simplicity, the residual action applies a six-dimensional wrench to the robot base, consisting of a three-dimensional force and a three-dimensional torque. The assisted MDP is given in Equation 2


$$\begin{array}{l} \hat{M}=\left(S,A\times \hat{A},\hat{P},r+\hat{r},\gamma \right), \end{array}$$ (2)

where an assisted action ${\tilde{\text{a}}}_{t}=\left({\text{a}}_{t},{\hat{\text{a}}}_{t}\right)\in A\times \hat{A}$ concatenates the joint-level and residual actions, $\hat{P}$ denotes the dynamics under residual-wrench assistance, and $\hat{r}$ denotes an exponential quadratic regularization reward on residual-wrench usage. This term discourages over-exploitation of assistance, since excessively large residual wrenches can become a non-physical stabilization channel rather than a temporary training scaffold.

Treating the residual action directly as a base wrench can produce abrupt force and torque commands, which may destabilize training. We therefore parameterize the residual wrench through a PD-like controller. Specifically, we add a residual policy alongside the normal actor network. The residual policy observes privileged information required for global motion tracking and outputs a residual action ${\hat{\text{a}}}_{t}$. Rather than treating ${\hat{\text{a}}}_{t}$ itself as a wrench, we interpret it as a desired base-position offset and a desired base-orientation offset expressed in exponential coordinates.

Both the actor policy and residual policy use MLPs with hidden sizes [512, 256, 128] and ReLU as activations for hidden layers. For the plasticity considerations discussed in Section 2.5, each hidden layer is followed by a LayerNorm layer. The residual policy additionally uses a Tanh output activation to bound residual actions and reduce over-exploitation of residual-wrench assistance.

Let *N* be the number of joints. Let the robot state at time *t* contain the base global position ${\text{p}}_{t}\in {\mathbb{R}}^{3}$, base global orientation **R**_*t*_∈SO(3), base global linear velocity ${\text{v}}_{t}\in {\mathbb{R}}^{3}$, base global angular velocity $\omega_{t}\in {\mathbb{R}}^{3}$, joint positions ${\text{q}}_{t}\in {\mathbb{R}}^{N}$, and joint velocities ${\overset{•}{\text{q}}}_{t}\in {\mathbb{R}}^{N}$. The corresponding reference quantities are denoted by ${\text{p}}_{t}^{⋆}$, ${\text{R}}_{t}^{⋆}$, ${\text{v}}_{t}^{⋆}$, $\omega_{t}^{⋆}$, ${\text{q}}_{t}^{⋆}$, and ${\overset{•}{\text{q}}}_{t}^{⋆}$. The scaling factor ρ controls the strength of residual assistance and is later reduced by the relaxation curriculum. Let $K_{p}^{f}$ and $K_{p}^{\tau }$ denote the stiffness for the residual force and torque PD controllers, let $K_{d}^{f}$ and $K_{d}^{\tau }$ denote the corresponding damping, and let ζ^*f*^ and ζ^τ^ denote the corresponding damping-ratio coefficients. The force vector **f**_*r, t*_ and torque vector ***τ***_*r, t*_ of the residual wrench are computed using Equations 3–6 as follows:


$$\begin{array}{ll} {\hat{\text{a}}}_{t} & =\left[\begin{array}{c} \delta_{p,t}\\ \delta_{\tau ,t} \end{array}\right]\in \hat{A}\subseteq {\mathbb{R}}^{6},\;\delta_{p,t},\delta_{\tau ,t}\in {\mathbb{R}}^{3}, \end{array}$$ (3)

$$\begin{array}{ll} {\text{R}}_{d,t} & ={\text{R}}_{t}^{⋆}\mathrm{Exp}\left(\delta_{\tau ,t}\right), \end{array}$$ (4)

$$\begin{array}{ll} {\text{f}}_{r,t} & =\rho K_{p}^{f}\left({\text{p}}_{t}^{⋆}+\delta_{p,t}-{\text{p}}_{t}\right)-K_{d}^{f}{\text{v}}_{t}, \end{array}$$ (5)

$$\begin{array}{ll} \tau_{r,t} & =\rho K_{p}^{\tau }{\text{R}}_{t}\mathrm{Log}\left({\text{R}}_{t}^{⊤}{\text{R}}_{d,t}\right)-K_{d}^{\tau }\omega_{t}. \end{array}$$ (6)

Here Exp:ℝ^3^ → SO(3) maps an exponential-coordinate vector to a rotation matrix, and Log:SO(3) → ℝ^3^ denotes the inverse map. All residual force and torque quantities are expressed in the world frame. The damping terms use the current base velocities rather than reference velocities for simulation stability.

The damping gains are coupled to the stiffness gains, as described in Equation 7


$$\begin{array}{l} K_{d}^{\chi }=\zeta^{\chi }\sqrt{\rho K_{p}^{\chi }},\;\chi \in \left\{f,\tau \right\}, \end{array}$$ (7)

where ζ^χ^ is tuned to keep the corresponding controller near critical damping throughout training. This coupling is important for stable training during residual relaxation.

The first stage trains the residual-wrench-assisted policy. Because this policy depends on external assistive wrenches, it is not physically feasible by itself. We therefore use it only to warm start the second stage, where residual assistance is reduced during training and disabled for final evaluation.

### 2.3 Relaxing external residual wrenches

The second stage gradually transfers the policy from the assisted MDP $\hat{M}$ back to the original MDP M. We enforce this transfer with a monotonic curriculum on the residual scaling factor ρ. As ρ decreases, the auxiliary wrench becomes weaker, and the policy must rely increasingly on physically feasible joint-level control. The assisted stage keeps the residual scale fixed at ρ = 1. During relaxation, each qualifying curriculum update additively decreases ρ by 5 × 10^−5^ until it reaches the lower bound $\rho_{\min}=10^{-4}$. This lower bound is used for training stability of the learnable residual policy. When ρ is driven exactly to zero, residual-policy actions no longer influence the dynamics or rewards, so the residual-policy gradient becomes uninformative and dominated by estimation noise. Maintaining a small nonzero residual scale preserves a weak action-reward coupling during relaxation, and helps with training stability. A reduction is triggered only when the maximum segment-wise early-termination failure rate falls below 0.05. Otherwise, ρ remains unchanged. This conservative update rule reduces the risk of policy collapse by delaying assistance removal until all motion segments are sufficiently stable. Because the curriculum is reduction-only, residual assistance is not restored after it has been reduced.

This relaxation process interacts with early termination. Early termination is commonly used in humanoid motion tracking to accelerate training and improve final performance. However, the corresponding thresholds are often manually specified (Peng et al., 2018; Luo et al., 2023). For simple motions, a fixed threshold can be sufficient. For highly dynamic and contact-rich motions, a threshold that is too tight may terminate dynamically feasible but temporarily inaccurate rollouts, whereas a threshold that is too loose may allow poor tracking behavior to dominate training. The appropriate threshold depends on motion difficulty and robot dynamics, both of which are hard to quantify and may vary across robots.

Starting from the residual-wrench-assisted policy allows the relaxation stage to use relatively strict termination bounds at the beginning. As residual assistance decreases, the termination bounds must also adapt so that the policy can continue learning from difficult segments instead of being terminated prematurely. This adaptive relaxation improves the final physically feasible policy.

### 2.4 Adaptive termination and sampling

During residual relaxation, we adapt the feasible bounds used by early termination according to motion difficulty. Rather than using a single global threshold, we partition each motion into *B* segments and maintain segment-wise threshold scales.

For each segment *b* and termination condition *c*, the active threshold is τ_*b, c*_ = η_*b, c*_τ_*c*_, where τ_*c*_ is the base threshold and η_*b, c*_ is initialized to 1. The three adapted conditions are base-height error, torso-orientation error, and end-effector height error. We maintain separate segment scales for each condition, so failures from one condition do not directly loosen the others. Each scale is clipped to condition-specific lower and upper bounds.

For each segment and condition, we estimate the failure rate ${\hat{p}}_{b,c}$ as the fraction of recent visits resolved to segment *b* that terminate due to condition *c*. Once enough recent visits are available, scales are updated with the rule described in Equation 8


$$\begin{array}{l} \eta_{b,c}\leftarrow \{\begin{array}{ll} \mathrm{clip}\left(\left(1+\alpha_{\text{relax}}\right)\eta_{b,c}\right), & {\hat{p}}_{b,c}>p_{\text{high}},\\ \mathrm{clip}\left(\left(1-\alpha_{\text{tighten}}\right)\eta_{b,c}\right), & {\hat{p}}_{b,c}<p_{\text{low}},\\ \eta_{b,c}, & \text{otherwise}. \end{array} \end{array}$$ (8)

Here *p*_high_ and *p*_low_ define a hysteresis band, and α_relax_ and α_tighten_ are small multiplicative update steps. This threshold curriculum is used only during the residual-relaxation stage. Unlike the residual-scale curriculum, this adaptive threshold update is not monotonic. A threshold can be tightened again after the policy improves on the corresponding segment.

We also use residual-wrench magnitude as a smooth proxy for dynamic difficulty when sampling reset frames. This proxy reflects the observation that more difficult motion segments tend to require larger residual wrenches during assisted tracking. The residual magnitude is ||**f**_*r, t*_||_2_+||***τ***_*r, t*_||_2_, averaged over environments currently in the segment. For each segment *b*, we maintain exponential moving averages *F*_*b*_ and *R*_*b*_ for failure counts and residual-wrench magnitude. The segment sampling weights are defined in Equation 9


$$\begin{array}{l} p_{b}\propto 0.5\frac{F_{b}+0.1/B}{\sum_{j}\left(F_{j}+0.1/B\right)}+0.5\frac{R_{b}+0.1/B}{\sum_{j}\left(R_{j}+0.1/B\right)}, \end{array}$$ (9)

The additive term 0.1/*B* is the uniform floor, which is added to every segment in both *F*_*b*_ and *R*_*b*_ before the two score vectors are normalized and mixed. This keeps segments with no recent failures or residual wrench sampleable, while the failure and residual terms bias reset states toward segments that are either unstable or require large assistance.

Table 1 summarizes the notation and settings that control the residual-wrench controller and relaxation curricula.

**Table 1:** Central notation and hyperparameters of RWARL.

Symbol	Definition	Setting
Residual-wrench controller		
${\hat{\text{a}}}_{t}$	Residual action encoding desired base-position and base-orientation offsets	ℝ^6^
**f**_*r, t*_, ***τ***_*r, t*_	Residual force and torque applied to the robot base	(ℝ^3^, ℝ^3^)
$K_{p}^{f}$, $K_{p}^{\tau }$	Stiffness gains for residual force and torque	(1000, 100)
ζ^*f*^, ζ^τ^	Damping-ratio coefficients in $K_{d}^{\chi }=\zeta^{\chi }\sqrt{\rho K_{p}^{\chi }}$, χ∈{*f*, τ}	(5, 0.1)
Residual-scale curriculum		
ρ	Residual-assistance scale at the start of training	1
ρ_min_	Training lower bound of the residual-assistance scale	10^−4^
Δρ	Residual-scale decrement per qualifying update	5 × 10^−5^
Adaptive termination		
*B*	Number of temporal segments per motion	*B* = ⌊*T*/*f*_*c*_⌋+1
τ_*c*_	Base threshold for termination condition *c*	(0.25m, 0.8, 0.25m)
η_*b, c*_	Segment-wise threshold scale for condition *c* (initial value; clipping ranges)	1;([1, 3], [1, 1.6], [1, 1.6])
*p*_high_, *p*_low_	Upper and lower bounds of the failure-rate hysteresis band	(0.05, 0.01)
α_relax_, α_tighten_	Threshold-relaxation and threshold-tightening steps	(0.05, 0.02)

Tuple values follow symbol order and, for condition-specific settings, base-height, torso-orientation, and end-effector-height order; *T* and *f*_*c*_ = 50Hz denote the number of control steps and control frequency.

### 2.5 Maintaining policy plasticity

Residual relaxation changes the environment dynamics observed by the policy. This distribution shift can reduce network plasticity and harm policy performance, potentially making continued training worse than training from scratch. To mitigate this issue, we apply shrink-and-perturb during training of the actor, critic, and residual actor networks. We also add layer-normalization layers after the linear layers of these networks. These choices are motivated by prior work on plasticity loss in online reinforcement learning, where LayerNorm and shrink-and-perturb-style regularization are studied as interventions under non-stationary on-policy training (Juliani and Ash, 2024).

## 3 Experiments

We evaluate whether residual-wrench-assisted training improves the tracking accuracy and successful-frame rate of humanoid motion tracking on highly dynamic and contact-rich reference motions. We first compare our method with a baseline humanoid tracking framework, then examine the coupling between residual-wrench relaxation and its supporting mechanisms, ablate the plasticity module and adaptive sampling, and finally summarize hardware experiments on Unitree G1.

### 3.1 Comparison with baseline

We use BeyondMimic (Liao et al., 2025) as the baseline and augment it with the residual-wrench mechanism described in Section 2. To isolate the effect of residual-wrench assistance, we keep the underlying motion-tracking framework fixed and compare the two methods under the same total number of training iterations.

We evaluate five reference motions from the AMASS (Mahmood et al., 2019) and LAFAN1 (Harvey et al., 2020) datasets. Figure 2 summarizes the motion set used in the comparison and ablation experiments. The set contains two highly dynamic motions with aerial phases, *jump twist* and *Webster flip*; two contact-rich motions, *helicopter* and *breakdance*; and one longer, relatively simple *dance* motion.

**Figure 2:**
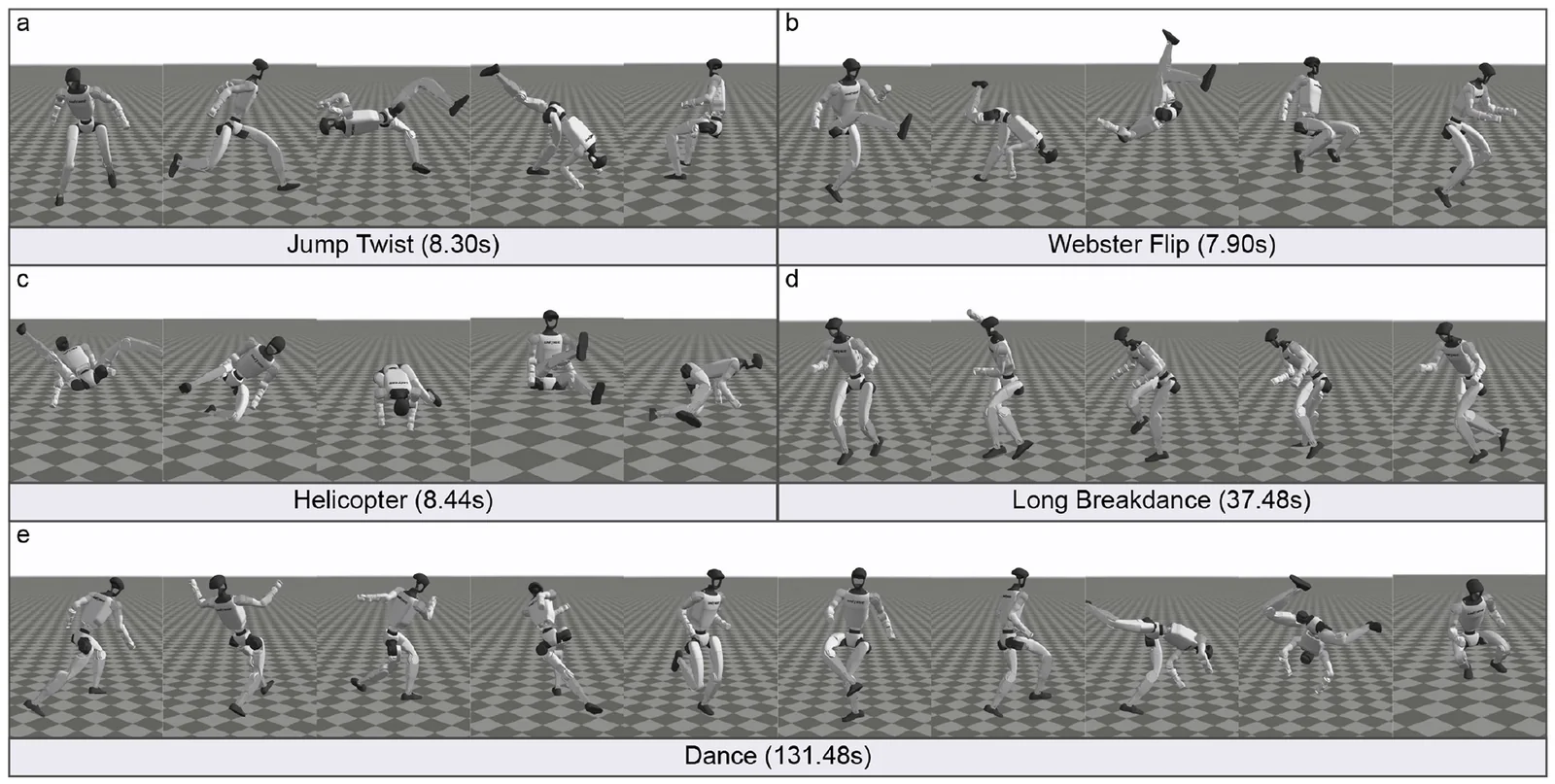
Reference motions used in the comparison and ablation experiments. Panels **(a, b)** show highly dynamic motions with aerial phases; panels **(c, d)** show contact-rich motions; and panel **(e)** shows a longer, relatively simple motion.

Instead of training a general multi-motion controller, we train a specialized policy for each motion. Each baseline policy is trained for 40,000 iterations. For our residual-wrench-assisted policies, stage 1 often converges quickly, so we allocate 5,000 iterations to stage 1 and 35,000 iterations to stage 2. The total training budget is therefore 40,000 iterations for each policy, exactly matching the baseline. This setup allows most motions to approach convergence under a fixed computational budget. All policies are trained on a single NVIDIA RTX 4090 GPU. We conduct all simulation experiments and policy training in NVIDIA Isaac Sim 4.5.0 and Isaac Lab 2.1.0. The simulation runs at 200Hz, and policies are updated at a control frequency of 50Hz. Each training iteration collects 24 control steps from each of 4,096 parallel environments, giving 98,304 transitions per iteration, and the maximum training episode duration is 10s. In RWARL, the residual action is updated together with the joint action at the control frequency of 50Hz, while the resulting PD wrench is recomputed from the current simulated state and applied at the simulation frequency of 200Hz. The baseline and RWARL use the same simulation and control frequencies, environment count, and rollout-batch configuration. We use the same training hyperparameters as BeyondMimic (Liao et al., 2025). Detailed observation, action, domain-randomization, reset-perturbation, and reward definitions are provided in the Supplementary data sheet S1.

Figure 3 shows the stage-2 relaxation process for the *breakdance* motion. At the beginning of stage 2, larger residual-wrench usage allows the policy to maintain low tracking error under the assisted dynamics, but the resulting behavior remains less physically feasible because it depends on external base assistance. As training proceeds, the residual-wrench magnitude decreases toward zero, transferring the policy toward the unassisted dynamics while preserving the low-error tracking behavior learned under the relaxed problem. By the end of relaxation, the policy requires only negligible residual-wrench assistance during training. All policy evaluations reported in the following experiments disable residual wrenches completely, so the reported metrics measure the unassisted joint-level policy.

**Figure 3:**
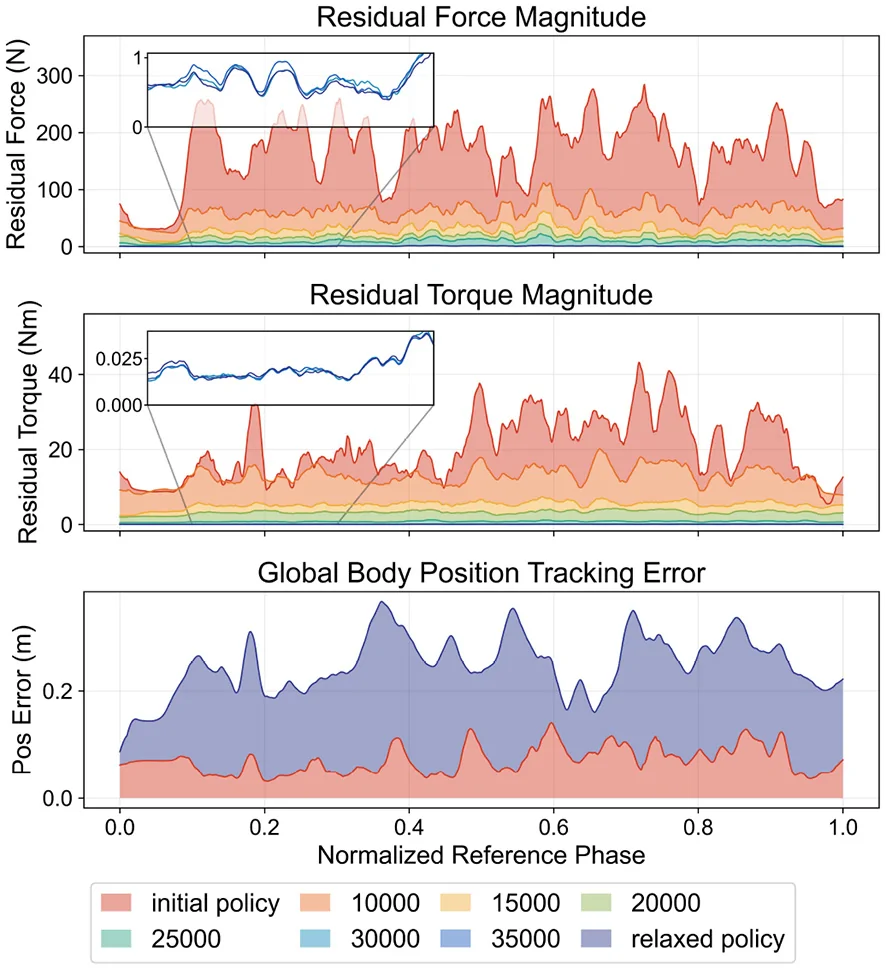
Stage-2 residual-wrench relaxation for the *breakdance* motion. Residual-wrench usage decreases toward zero while tracking error remains low, and final evaluations disable residual wrenches completely.

For each policy, we collect 256 rollouts that start from the first frame of the reference trajectory and end at the final frame. To ensure a fair comparison, all policies tracking the same motion use the same set of initial states, including the robot base pose, base velocity, joint positions, and joint velocities. We report successful-frame rates and tracking accuracy for each policy in Table 2. Successful frames are identified by applying the same failure checks used for early termination during training to the recorded rollout trajectory. During evaluation, these checks do not stop rollouts when triggered. Instead, each rollout is collected until the reference trajectory reaches its final frame, and we identify the first frame at which any failure condition is triggered. Let **x**_*t*_ denote the policy-induced robot state and **g**_*t*_ denote the corresponding reference state. A frame is marked as failed, together with all subsequent frames in that rollout, if any of the following conditions has been triggered at timestep *t*:


$$\begin{array}{cc} \text{base height: } & |h_{\text{base}}\left({\text{x}}_{t}\right)-h_{\text{base}}\left({\text{g}}_{t}\right)|>0.75\text{m},\\ \text{torso projected gravity: } & |\eta_{z,\text{torso}}\left({\text{x}}_{t}\right)-\eta_{z,\text{torso}}\left({\text{g}}_{t}\right)|>1.28,\\ \text{ankle height: } & \underset{e\in E_{\text{ankle}}}{\max}|h_{e}\left({\text{x}}_{t}\right)-h_{e}\left({\text{g}}_{t}\right)|>0.40\text{m}. \end{array}$$

Here, *h*_base_ denotes the base height, η_*z*, torso_ denotes the vertical component of projected gravity in the torso frame, and *h*_*e*_ denotes the vertical position of end-effector *e*. The set $E_{\text{ankle}}$ contains the left and right ankle end-effectors. We define the successful-frame rate as the number of frames before the first failure event, aggregated across all rollouts, divided by the total number of recorded frames. For tracking accuracy, we report root mean squared error (RMSE) metrics for joint position, global base position, global base orientation, global body position, and global body orientation. Tracking errors are averaged over the same successful frames before the first failure event in each rollout. This excludes post-failure frames from the tracking-error evaluation because they often produce large, uninformative errors that can bias the comparison of tracking accuracy.

**Table 2:** Tracking accuracy comparison between the baseline and our method.

Motion	Method	*E*_Joint_↓	*E*_Base Pos._↓	*E*_Base Ori._↓	*E*_Body Ori._↓	*E*_Body Pos._↓	Success-frame rate ↑
jump twist	Baseline	0.147	0.158	0.173	0.254	0.172	57.84%
	Ours	0.128	0.096	0.206	0.193	0.107	99.61%
	Δ	**−12.9%**	**−38.9%**	+19.3%	**−24.1%**	**−37.8%**	**+41.8**
Webster flip	Baseline	0.159	0.102	0.246	0.230	0.115	99.63%
	Ours	0.158	0.084	0.247	0.232	0.097	99.65%
	Δ	**−0.7%**	**−17.7%**	+0.3%	+0.5%	**−15.7%**	**+0.0**
helicopter	Baseline	0.174	0.112	0.245	0.229	0.126	99.40%
	Ours	0.146	0.105	0.270	0.221	0.115	98.91%
	Δ	**−16.4%**	**−6.1%**	+10.1%	**−3.5%**	**−8.6%**	−0.5
breakdance	Baseline	0.246	0.170	0.386	0.410	0.182	98.07%
	Ours	0.234	0.133	0.302	0.344	0.146	98.58%
	Δ	**−5.0%**	**−21.6%**	**−21.8%**	**−16.3%**	**−19.7%**	**+0.5**
dance	Baseline	0.098	0.098	0.120	0.149	0.103	99.81%
	Ours	0.107	0.092	0.125	0.161	0.099	99.49%
	Δ	+8.9%	**−5.7%**	+3.8%	+8.4%	**−4.5%**	−0.3

Each error is reported as a root mean square error (RMSE) over successful frames. *E*_Joint_ measures joint-position tracking error. *E*_Base Pos._ and *E*_Base Ori._ measure the global position and orientation errors of the robot base. *E*_Body Ori._ and *E*_Body Pos._ measure the global orientation and position errors of robot bodies. Lower error values indicate better tracking accuracy. For RMSE metrics, Δ denotes the relative percentage change from the baseline to our method. For successful-frame rate, Δ denotes the percentage-point difference. Bold values indicate improvements of our method over the baseline, with negative relative changes in RMSE and positive percentage-point changes in successful-frame rate.

Table 2 shows promising overall tracking performance for the final RWARL policies, with lower or comparable errors in most metrics. However, the increases in base-orientation RMSE for *jump twist* and *helicopter* are exceptions. These exceptions mainly reflect differences in the selected pre-terminal frames rather than a general degradation in RWARL tracking performance.

We compute the RMSE values in Table 2 separately for each policy over frames before the first terminal event in each rollout, so different termination times produce different evaluation supports. Take the *jump twist* motion as an example: 57.84% of the baseline rollout frames are pre-terminal, compared with 99.61% for RWARL. As a result, the RWARL error includes later motion phases that are absent from the baseline metric. When both policies are evaluated on the same support, using matched trial–frame pairs before either policy's first terminal event, the results change. Base-orientation RMSE decreases from 0.163 to 0.152 (–6.66%) for *jump twist* and increases only from 0.246 to 0.252 (+2.53%) for *helicopter*. These matched results show that frame selection accounts for most of the 19.3% and 10.1% increases reported in Table 2.

The matched-support calculation isolates the frame-selection effect. Table 2 is therefore the more demanding evaluation and provides a broader view of overall tracking performance because it evaluates each policy over its entire achieved pre-terminal horizon. Across this full horizon, RWARL still has an overall tracking advantage over the baseline for both motions, with lower RMSE in four of the five tracking metrics.

The results show that residual-wrench-assisted training generally improves global-position and body-position tracking, with the largest gains appearing on motions that contain difficult transitions. Figure 4 visualizes two representative cases, comparing the baseline with our final unassisted RWARL policy. Supplementary video S1 provides the full visual comparison between the baseline policy and our final unassisted RWARL policy. In the turning-around motion, the baseline turns by only a small angle and then stops rotating, whereas our method follows the reference through the full turn. In the cross-step motion, the baseline adopts a more conservative strategy that keeps both feet close to the ground, while our method reproduces the dynamically feasible leg-crossing behavior in the reference motion. These behaviors may be dynamically feasible but hard to discover because they lie behind sparse feasible transition regions. Residual-wrench assistance improves exploration in these regions during training, which can lead to more accurate physically feasible tracking after relaxation.

**Figure 4:**
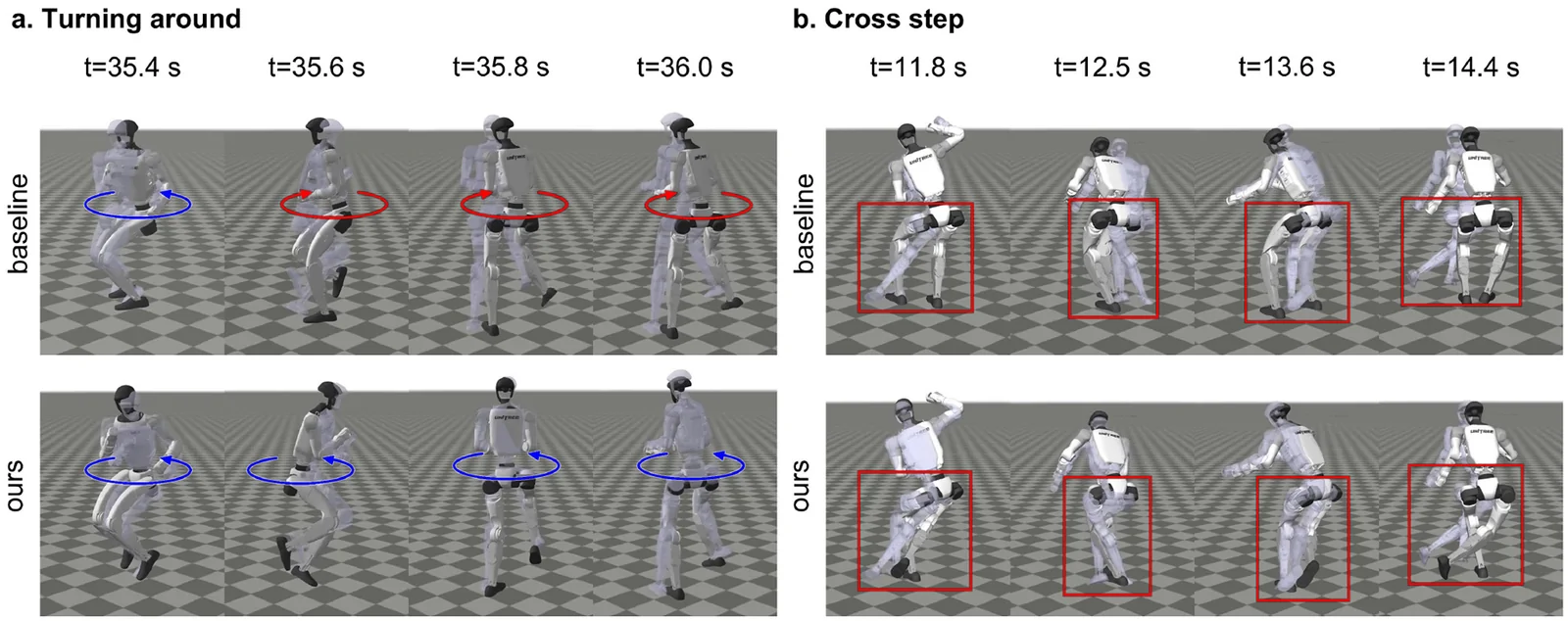
Visual comparison of subtle behaviors tracked by the baseline policy and our relaxed policy on Unitree G1 under the same training budget. The turning-around and cross-step examples in panels **(a, b)**, respectively, are phases of the evaluated *breakdance* reference motion. The transparent robot shows the reference motion. In the turning-around motion **(a)**, the baseline policy rotates by only a small angle and then stops rotating, whereas our policy follows the reference through the full turn. Red circular arrows in panel **(a)** denote rotation directions that are inconsistent with the reference, while blue circular arrows denote rotation directions that are consistent with the reference. In the cross-step motion **(b)**, the baseline policy tends to keep both feet near the ground, whereas our policy tracks the reference leg-crossing behavior.

The improvement is not uniform across all motions. In Table 2, the *dance* motion shows little improvement relative to the baseline. One possible explanation is that this motion contains fewer low-reachability transitions, so the baseline already samples and optimizes the relevant states sufficiently well. In such cases, the residual-wrench scaffold provides less additional benefit.

### 3.2 Predecessor-state reachability of critical states

Beyond the overall tracking performance, we examine a possible mechanism behind the improvements on difficult transitions. Residual-wrench-assisted training may change the closed-loop state distribution so that the policy reaches predecessor states from which critical reference states are easier to track.

Reference trajectories can contain phases with different dynamic feasibility margins. Some motions have narrow dynamically feasible regions around specific reference states; for example, Ma et al. (2021) suggests that the takeoff state is crucial for the backflip motion. We hypothesize that residual-wrench-assisted training helps the policy reach predecessor states that are more favorable for tracking these critical phases, thereby improving closed-loop robustness.

To test this hypothesis, we compare the baseline policy with our relaxed policy. If residual-wrench-assisted training mainly helps on difficult transitions, the largest error gaps between the two policies should identify phases where the baseline fails to reach a favorable closed-loop state. We define *critical states of the reference motion* as reference states where the baseline policy has substantially higher tracking error than our policy. We then evaluate whether predecessor states at fixed temporal offsets before these critical phases lead to successful closed-loop tracking of the corresponding reference-state neighborhood.

We instantiate this diagnostic on the *breakdance* motion, where the baseline and our relaxed policy both achieve high overall successful-frame rates but differ noticeably around difficult transitions. We select seven critical phases where the baseline has substantially larger tracking error than our relaxed policy. For each critical phase, we roll out the policy from predecessor states at offsets of 0.25s, 0.50s, and 1.00s before the selected phase. Each phase–predecessor condition is evaluated with 16 rollouts under independently sampled observation noise, producing different closed-loop trajectories. We count a rollout as a hit if it reaches a target neighborhood around the selected reference state within a 0.2s target window, using a mean body-position error threshold of 0.25m and a mean body-orientation error threshold of 1.0rad.

To further separate predecessor-state quality from controller capability, we also evaluate both controllers from matched exported predecessor-state distributions. For each offset, we export natural predecessor states from the baseline and from our relaxed policy, and also test predecessor states sampled directly from the reference motion at the same phase. We use trained-policy rollouts rather than direct measurements of training-time visitation density, so it evaluates the reachability mechanism through closed-loop behavior. Figure 5 summarizes the diagnostic. Figure 5a shows the global body-position tracking error profiles used to select the seven critical states. Figure 5b reports the cross-policy reachability matrix: rows specify the predecessor offset, columns specify the predecessor-state source, and the two heatmaps compare the baseline controller with our relaxed controller. Figure 5c compresses the matrix into two hit-rate contrasts, separating the effect of predecessor-state source from the effect of rollout controller. Each heatmap cell uses 112 rollouts, corresponding to seven critical phases and 16 noisy rollouts per phase.

**Figure 5:**
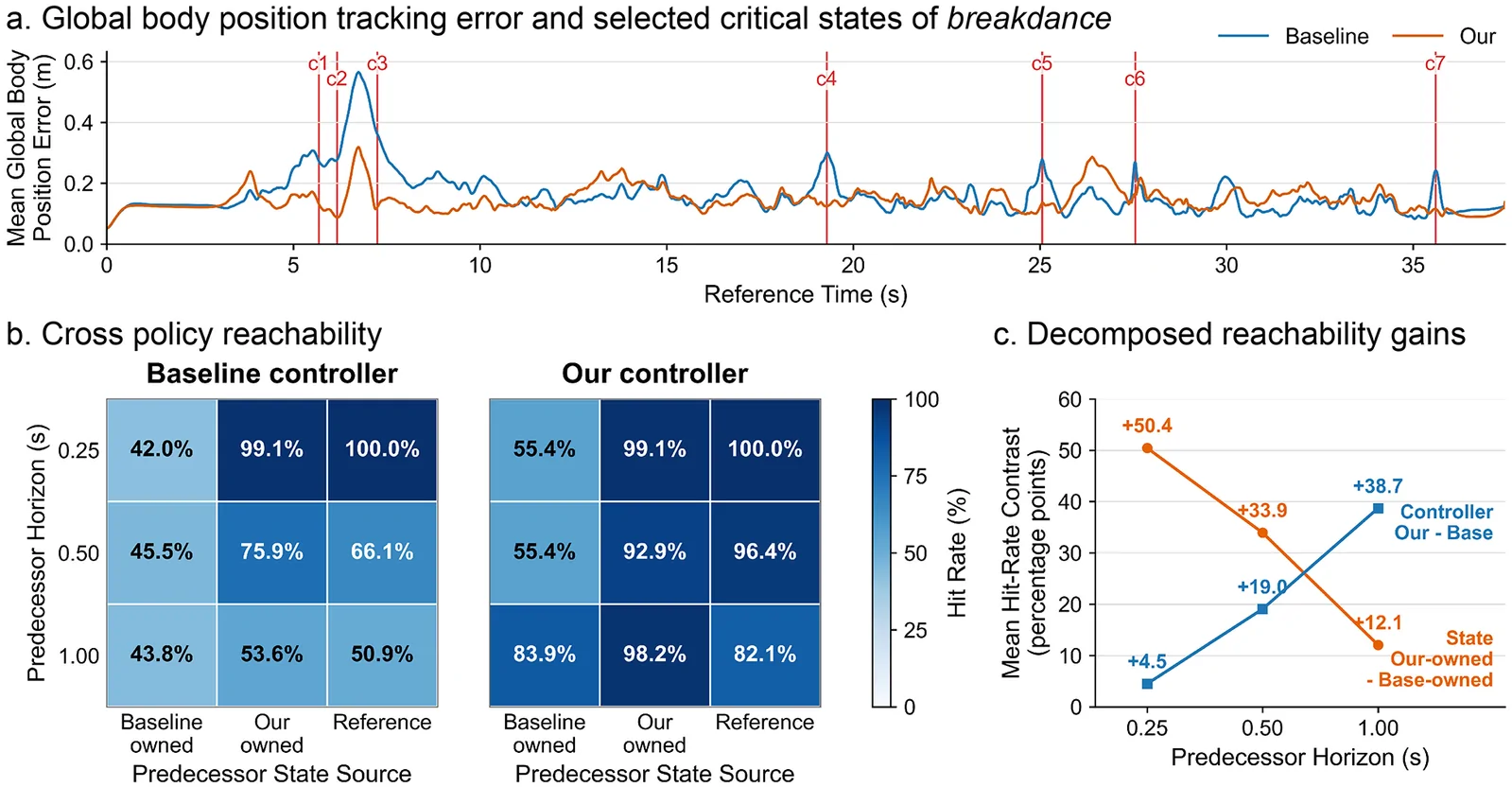
Cross-policy predecessor-state diagnostic on selected critical states of the *breakdance* motion. **(a)** Mean global body-position tracking error over reference time for the baseline and our relaxed policy. Red markers indicate the seven critical states used in the diagnostic, selected from phases where the baseline has substantially larger tracking error than our policy. **(b)** Cross-policy reachability matrix for predecessor horizons of 0.25 s, 0.50 s, and 1.00 s. Each heatmap entry reports the fraction of rollouts that reach the selected reference-state neighborhood when either the baseline controller or our controller is initialized from baseline-owned, ours-owned, or reference predecessor-state distributions. **(c)** Decomposed reachability gains computed from the matrix in panel **(b)**. The orange curve measures the predecessor-state contrast between ours-owned and baseline-owned predecessor states, averaged over both controllers. The blue curve measures the controller contrast between our controller and the baseline controller, averaged over all predecessor-state distributions.

Figure 5 supports three observations. First, Figure 5b shows that ours-owned predecessor states consistently lead to higher hit rates than baseline-owned predecessor states for both controllers and all offsets. The orange curve in Figure 5c summarizes this predecessor-state contrast, which is largest at short horizons and remains positive at 1.00s. This suggests that residual-wrench-assisted training reaches predecessor states from which a policy can more easily track the selected critical states. Second, Figure 5b shows that reference-motion predecessor states are not always the easiest starting points for closed-loop tracking. At 0.50s, the baseline controller reaches the target neighborhood more often from ours-owned predecessors than from reference-motion predecessors; at 1.00s, the same pattern appears for both controllers. This suggests that some reference predecessor states may be less dynamically compatible with the controller than predecessor states naturally reached by our method. Third, controller recovery ability becomes more visible at longer offsets, as shown by the increasing blue curve in Figure 5c. At 1.00s, our relaxed controller achieves higher hit rates than the baseline from all tested predecessor-state distributions, although predecessor-state quality still remains influential. Together, these results ground the exploration explanation in trained-policy behavior: residual-wrench-assisted training leads to predecessor-state distributions and controller recovery behavior that better support tracking of critical reference states.

We repeat the complete diagnostic on *Webster flip*, a highly dynamic motion with an aerial phase. We select five critical phases using the same tracking-error-gap criterion and retain the same predecessor horizons, rollout count per phase, target window, and hit thresholds used for *breakdance*. Each heatmap cell therefore uses 80 rollouts, corresponding to five critical phases and 16 noisy rollouts per phase. Figure 6 reports the tracking-error profile, cross-policy reachability matrix, and decomposed hit-rate contrasts.

**Figure 6:**
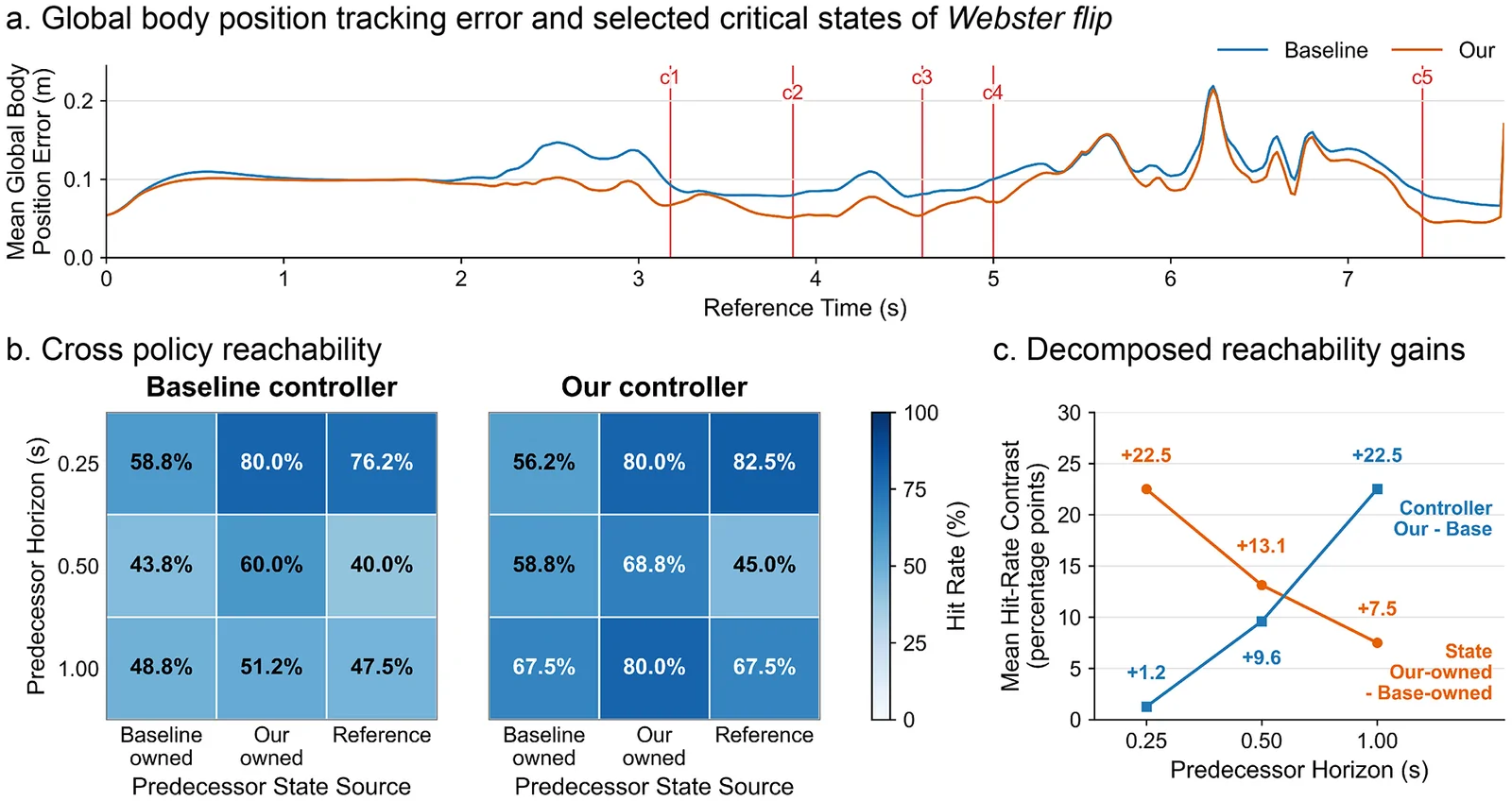
Cross-policy predecessor-state diagnostic on five selected critical states of the *Webster flip* motion. The protocol and panel definitions match Figure 5; each heatmap cell contains 80 rollouts. **(a)** Global body-position tracking error and selected critical states of *Webster flip*. **(b)** Cross policy reachability. **(c)** Decomposed reachability gains.

Figure 6 shows the same qualitative decomposition as the *breakdance* diagnostic: across the tested horizons, the predecessor-state contrast decreases from 22.5 to 7.5 percentage points, while the controller contrast increases from 1.2 to 22.5 points. Overall, the two diagnostics are consistent with the interpretation that the proposed training scheme shapes predecessor-state reachability around critical reference states in both contact-rich and aerial motions.

### 3.3 Residual-wrench relaxation without supporting mechanisms

RWARL is organized around residual-wrench relaxation. Residual-wrench assistance defines the relaxed tracking problem in stage 1 and the transition toward the original unassisted dynamics in stage 2. Adaptive termination, residual-wrench-aware sampling, and the plasticity module support this transition by managing the changing termination bounds, training distribution, and policy adaptability as assistance decreases. RWARL therefore forms a coupled training pipeline: the residual wrench defines the relaxation path, while the supporting mechanisms help the policy follow that path toward unassisted control.

To examine residual-wrench relaxation without these supporting mechanisms, we train a residual-wrench-only variant for the same 40,000-iteration budget. This variant adds the residual-wrench controller and residual-scale curriculum to the baseline, while disabling adaptive termination and the plasticity module. Because the baseline already uses failure-driven adaptive sampling, the variant retains this sampler but omits RWARL's residual-wrench-magnitude sampling term.

After 40,000 iterations, residual relaxation remains incomplete for *jump twist* (ρ = 0.9120) and *breakdance* (ρ = 0.4565 and still decreasing). Because both checkpoints retain substantial residual assistance, we do not evaluate them as unassisted policies.

Table 3 summarizes the relaxation outcomes for all five motions and reports evaluation changes for the three motions on which the residual-wrench-only variant completes relaxation. These final policies are evaluated with the same initial-state manifests as in Table 2. For the three completed policies, residual-wrench-only training lowers the successful-frame rate relative to the baseline. The residual-wrench-only policies also exhibit generally higher tracking errors across the three motions. Thus, completing the relaxation schedule alone does not ensure that the policy retains its tracking quality as residual assistance is removed.

**Table 3:** Residual-wrench-only ablation relative to the baseline.

Motion	Relaxation	*E*_Joint_↓	*E*_Base Pos._↓	*E*_Base Ori._↓	*E*_Body Ori._↓	*E*_Body Pos._↓	Success-frame rate ↑
Jump twist	ρ = 0.9120	–	–	–	–	–	–
Webster flip	Complete	+14.1%	+31.9%	+7.5%	+21.3%	+32.0%	−0.26
Helicopter	Complete	**−4.1%**	+36.9%	+4.5%	+7.4%	+31.7%	−0.44
Breakdance	ρ = 0.4565	–	–	–	–	–	–
Dance	Complete	+21.7%	+51.3%	+14.3%	+27.2%	+51.6%	−0.95

“Complete” indicates that the residual scale ρ reached its training lower bound within 40,000 iterations; otherwise, the Relaxation column reports the value of ρ at the end of training. Metrics are reported only for policies that completed relaxation. The Successful-Frame Rate column reports the residual-wrench-only policy's rate minus the baseline rate in percentage points. Each error column reports the relative RMSE change from the baseline to the residual-wrench-only policy, so a negative value indicates lower error. Bold values indicate improvements achieved by the residual-wrench-only policy relative to the baseline.

The two incomplete schedules reveal the bottleneck directly addressed by adaptive termination. Under fixed termination bounds, tracking on *jump twist* and *breakdance* does not become reliable enough for residual assistance to keep decreasing, leaving substantial assistance after 40,000 iterations. Adaptive termination targets this bottleneck by adjusting the segment-wise termination bounds according to observed failures as assistance decreases. The full RWARL pipeline incorporates adaptive termination and completes relaxation on all five motions.

The regressions on the three policies that reach the minimum residual scale reveal a second bottleneck: completing the scale schedule alone does not preserve tracking quality relative to the baseline. This pattern is consistent with the adaptation challenge posed by the changing dynamics and training distribution, which the plasticity module and residual-wrench-aware sampling are designed to address. The full RWARL pipeline coordinates these mechanisms so that the policy can both progress along the residual-relaxation path and retain useful tracking behavior at its unassisted endpoint.

### 3.4 Ablation of plasticity module

Residual-wrench-assisted training changes the environment dynamics experienced by the policy during relaxation. This distribution shift can reduce network plasticity and degrade policy performance. To mitigate this effect, we adopt two techniques that have been shown to improve plasticity in on-policy reinforcement learning (Juliani and Ash, 2024): adding LayerNorm after Linear layers and applying shrink-and-perturb. We ablate these methods using the same successful-frame-rate and tracking-accuracy metrics as in the baseline comparison.

Table 4 shows that the policy with the plasticity module improves policy performance in most comparisons against the policy without the plasticity module. Some motions also show large reductions in tracking error, especially for the longer reference trajectories. For example, adding the plasticity module reduces base-position and body-position errors by 30.9% and 29.5% on the 37.48s *breakdance* motion, and by 43.9% and 42.4% on the 131.48s *dance* motion.

**Table 4:** Plasticity-module ablation on successful frames.

Motion	Method	*E*_Joint_↓	*E*_Base Pos._↓	*E*_Base Ori._↓	*E*_Body Ori._↓	*E*_Body Pos._↓	Success-frame rate ↑
Jump twist (8.30s)	w/o PM	0.138	0.109	0.222	0.223	0.120	99.69%
	w/ PM	0.128	0.096	0.206	0.193	0.107	99.61%
	Δ	**−7.0%**	**−11.4%**	**−7.2%**	**−13.4%**	**−11.1%**	−0.1
Webster flip (7.90s)	w/o PM	0.161	0.087	0.264	0.236	0.102	99.47%
	w/ PM	0.158	0.084	0.247	0.232	0.097	99.65%
	Δ	**−2.3%**	**−3.3%**	**−6.4%**	**−1.9%**	**−4.8%**	**+0.2**
Helicopter (8.44s)	w/o PM	0.142	0.105	0.244	0.200	0.115	99.47%
	w/ PM	0.146	0.105	0.270	0.221	0.115	98.91%
	Δ	+3.0%	**−0.2%**	+10.6%	+10.7%	**−0.1%**	−0.6
Breakdance (37.48s)	w/o PM	0.229	0.193	0.330	0.337	0.207	97.07%
	w/ PM	0.234	0.133	0.302	0.344	0.146	98.58%
	Δ	+2.4%	**−30.9%**	**−8.5%**	+2.1%	**−29.5%**	**+1.5**
Dance (131.48s)	w/o PM	0.123	0.164	0.133	0.182	0.171	97.30%
	w/ PM	0.107	0.092	0.125	0.161	0.099	99.49%
	Δ	**−13.1%**	**−43.9%**	**−6.3%**	**−11.5%**	**−42.4%**	**+2.2**

The table reports the same tracking metrics as Table 2, but excludes the baseline because this ablation isolates the effect of the plasticity module. PM denotes the plasticity module. w/ PM denotes our method with the plasticity module. We use the same w/ PM rollout data as in the baseline comparison experiments, providing a shared reference for the plasticity ablation. w/o PM denotes the ablated policy trained without the plasticity module. The duration shown below each motion name denotes the length of the reference trajectory. Lower error values indicate better tracking accuracy. For RMSE metrics, Δ denotes the relative percentage change from w/o PM to w/ PM. For successful-frame rate, Δ denotes the percentage-point difference. Bold values indicate improvements with the plasticity module.

These improvements do not hold for every motion: on the 8.44s *helicopter* motion, the policy with the plasticity module reduces body-position error only marginally and degrades joint error, orientation errors, and successful-frame rate. This exception is consistent with the regularization tradeoff in plasticity-preserving methods: shrink-and-perturb biases weights toward smaller magnitudes, while LayerNorm constrains intermediate activation statistics (Juliani and Ash, 2024; Dohare et al., 2024). Such constraints help preserve adaptability under distribution shift, but prior plasticity studies also show that their effect can be task- and setting-dependent, and poorly matched interventions can reduce performance (Juliani and Ash, 2024; Dohare et al., 2024). For short reference motions where stage-2 training can already adapt sufficiently, the plasticity module may therefore add constraints whose marginal benefit is small or negative.

Overall, the ablation indicates that the plasticity module is most useful for longer motions, where maintaining adaptability during residual-wrench relaxation appears more important.

### 3.5 Ablation of adaptive sampling

We ablate adaptive sampling to examine whether focusing training on difficult motion segments is itself sufficient to improve tracking. The results in Table 5 show that adaptive sampling is not uniformly beneficial for tracking accuracy or successful-frame rate. For each evaluated motion, we collect four rollout groups: the baseline trained without adaptive sampling, the baseline trained with adaptive sampling, our method trained without adaptive sampling, and our method trained with adaptive sampling.

**Table 5:** Adaptive-sampling ablation.

Motion	Policy	*ΔS*_Frame_ (pp)	*ΔE*_Joint_	*ΔE*_Base Pos._	*ΔE*_Base Ori._	*ΔE*_Body Ori._	*ΔE*_Body Pos._
Webster flip	Baseline	−0.19	+5.9%	+8.7%	+1.7%	+5.7%	+9.0%
	Ours	−0.16	+7.4%	+0.3%	**−1.6%**	+2.7%	+1.2%
Break dance	Baseline	−0.79	+9.7%	+45.2%	+22.4%	+28.0%	+40.1%
	Ours	**+1.80**	**−3.7%**	**−16.2%**	**−7.8%**	**−11.3%**	**−12.9%**

AS denotes adaptive sampling. The table compares each policy trained w/ AS against its counterpart trained w/o AS. Δ*S*_Frame_ is the absolute successful-frame-rate difference in percentage points, computed as *S*_Frame, w/ AS_−*S*_Frame, w/o AS_. Each tracking metric is an RMSE over successful frames, and Δ*E* is the relative error change from w/o AS to w/ AS, computed as (*E*_w/ AS_−*E*_w/o AS_)/*E*_w/o AS_. Negative Δ*E* indicates lower tracking error with AS. Bold values indicate improvements from enabling adaptive sampling relative to disabling it for the same policy.

For the baseline on *breakdance*, enabling adaptive sampling worsens all reported successful-frame RMSE aggregates. The largest degradations are in global position tracking, where base-position RMSE increases by 45.2% and body-position RMSE increases by 40.1%. For *Webster flip*, the effect is much smaller: the baseline successful-frame rate decreases by only 0.19 percentage points, and the RMSE changes remain within 9.0%.

These results indicate that the effect of adaptive sampling depends on both the policy formulation and the difficulty of the tracked motion. On *breakdance*, the same adaptive-sampling mechanism hurts the baseline but helps our residual-wrench policy, raising the successful-frame rate from 96.78% to 98.58% and improving all reported successful-frame RMSE aggregates. On *Webster flip*, however, our policy already reaches a 99.80% successful-frame rate without adaptive sampling, and enabling adaptive sampling reduces this rate only slightly, to 99.65%, while producing only minor RMSE changes compared with *breakdance*. This contrast suggests that adaptive sampling has larger marginal value when the policy still struggles to traverse difficult segments, but it can provide little benefit when the policy is already stable.

One possible explanation is that adaptive sampling changes the effective training distribution. Oversampling difficult segments increases exposure to rare failures, which can help when those segments are the limiting factor, as in our *breakdance* policy. However, when the policy or dynamics model cannot exploit this biased distribution, the same mechanism may over-concentrate optimization on hard segments and degrade average tracking over successful frames. Based on these observations, we keep adaptive sampling in our full method: it improves our residual-wrench policy on the more difficult *breakdance* motion, while introducing only minor changes when performance is already saturated on *Webster flip*.

### 3.6 Hardware experiments

We deploy the final unassisted RWARL policies on Unitree G1 hardware to evaluate whether the behaviors learned through residual-wrench relaxation remain physically executable on the real robot. Because each RWARL policy is trained on a single motion clip, hardware execution requires a separate controller to maintain balance before the clip starts and after the clip ends. We use SONIC (Luo et al., 2026) for these transitions, passing control to the corresponding RWARL policy for the target clip and back to SONIC afterward. For reference motions that end in postures far from nominal standing, SONIC does not provide a native recovery behavior back to standing, so we do not evaluate post-motion recovery and allow the robot to fall after the tracked clip ends.

The hardware policies are deployed with a C++ runtime to reduce control latency. Some observations used in simulation, such as base linear velocity, are not directly available during hardware deployment. Rather than introducing a state estimator, we train the hardware-deployed policies without these inaccessible observations. All other training settings are kept identical to the simulation experiments. Figure 7 shows representative hardware executions for highly dynamic motions with aerial phases and for contact-rich motions. Supplementary video S2 shows the corresponding hardware experiments on Unitree G1. These deployments show that the relaxed policies can track challenging references on hardware after residual assistance has been removed.

**Figure 7:**
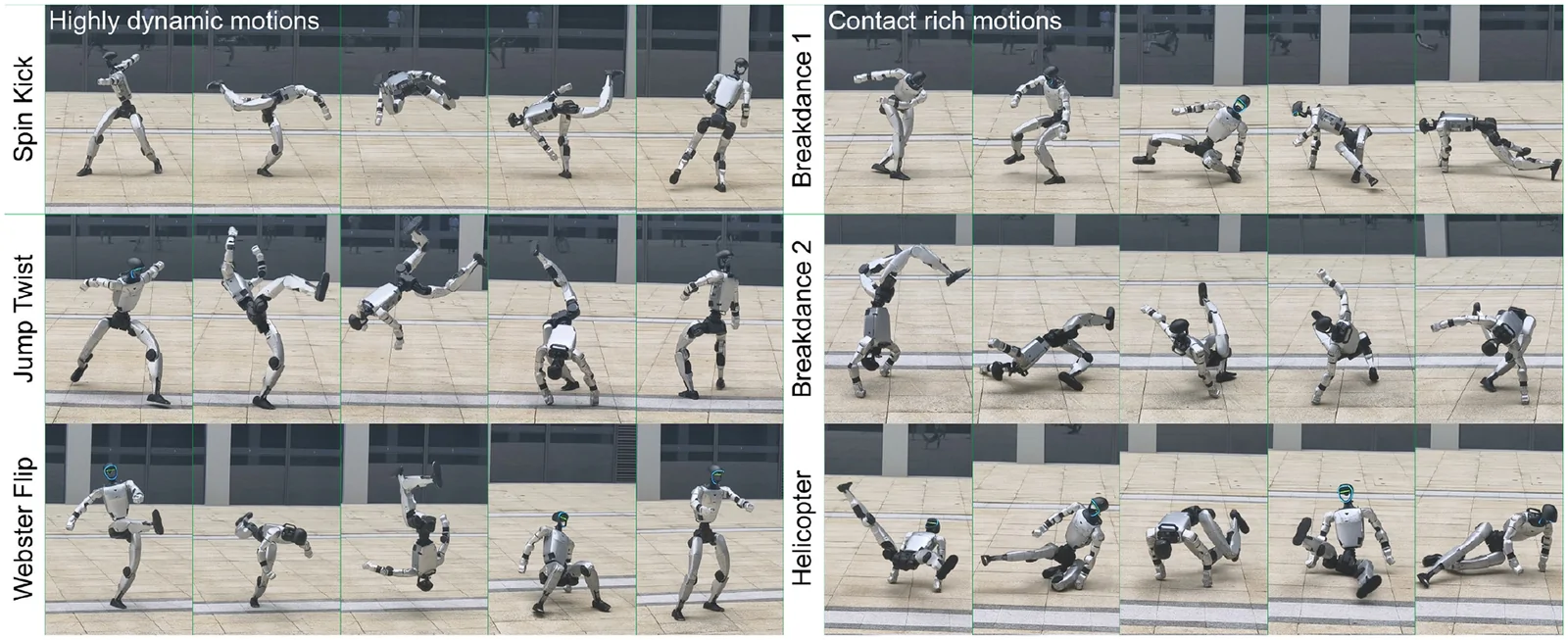
Hardware deployment of final unassisted RWARL policies on Unitree G1. The left column shows highly dynamic motions with aerial phases, and the right column shows contact-rich motions, with three representative examples in each category. All deployments use policies obtained after stage-2 relaxation, showing that residual-wrench-assisted training can recover physically feasible behaviors that transfer to hardware.

## 4 Discussion and conclusion

In this paper, we propose RWARL, which uses residual wrenches as a temporary training scaffold for humanoid motion tracking. Instead of training a tracking controller from scratch, the method first learns under a relaxed assisted MDP and then gradually anneals the residual assistance to recover a physically feasible policy. The experiments indicate that this procedure improves tracking accuracy on the evaluated highly dynamic and contact-rich motions, with the largest gains appearing in motion phases where the baseline exhibits comparatively large tracking errors.

The results support the interpretation that residual-wrench assistance improves exploration during training, even though the final policy is evaluated without residual wrenches. During the assisted stage, residual forces and torques are applied to the robot base in addition to the joint-level controller. This assistance allows rollouts to visit states near critical reference phases that are not consistently reached by the baseline policy. After relaxation, the policy no longer uses residual wrenches, but the training process can still leave it with better predecessor-state distributions around critical phases. The cross-policy predecessor-state diagnostic is consistent with the explanation that predecessor states collected from the relaxed RWARL policy more often lead to successful tracking of selected critical states than predecessor states collected from the baseline policy. Thus, the main benefit of RWARL appears to come from shaping the training distribution around reference phases where tracking is difficult and errors are large.

The residual-assistance strategy creates a tradeoff between reachable-state coverage and dynamics fidelity. Larger residual assistance makes more states practically reachable during training, but it also moves the assisted dynamics farther from the unassisted robot dynamics that the final policy must use. The relaxation stage must therefore recover physical fidelity while preserving the useful behaviors discovered under assistance. If the residual scale ρ is reduced too quickly, the reachable training distribution and the transition dynamics can change abruptly before the policy has adapted to the less assisted environment. This distribution shift can reduce policy plasticity and degrade continued training, especially on longer motions. Our ablation suggests that LayerNorm and shrink-and-perturb can mitigate this issue in some settings (Juliani and Ash, 2024; Dohare et al., 2024), but these mechanisms do not remove the need for a careful relaxation schedule.

The method is also most useful when the reference motion contains transitions where the tracking errors are large. For less dynamic motions, or motions where the baseline already reaches near-saturated tracking performance, residual assistance provides less additional benefit. In those cases, the extra stage required to anneal the residual wrench can delay the point at which the policy becomes directly deployable. The method therefore trades additional training overhead for improved exploration on motion segments that dominate baseline tracking error, rather than providing a uniform improvement for all reference motions.

Future work could make residual relaxation more adaptive and motion-aware. One direction is to condition the tracking policy on the residual-assistance scale ρ or on a learned dynamics embedding, so that the policy can adapt smoothly across the assisted and unassisted dynamics encountered during relaxation. Such a dynamics-conditioned policy could support an adaptive schedule that reduces ρ according to predicted performance degradation, failure rate, or uncertainty, instead of relying on a conservative hand-designed schedule. Another direction is to predict the expected marginal benefit of residual assistance before or during training. Such a predictor could identify motions and motion segments that are likely to benefit from residual-wrench assistance, allowing the method to focus computation on high-error or contact-rich phases while avoiding unnecessary relaxation overhead on motions that are already easy to track.

Overall, the results suggest that residual wrenches are useful as an optimization scaffold for humanoid tracking problems with highly dynamic or contact-rich reference motions. They broaden the set of useful states encountered during training and can help the final unassisted policy track high-error phases more accurately. At the same time, the scaffold changes the training dynamics and must be removed carefully. Improving this relaxation process is therefore central to making residual-wrench-assisted training more efficient and broadly applicable.

## Data Availability

The original contributions presented in the study are included in the article/Supplementary material, further inquiries can be directed to the corresponding authors.
