# Supplementary material for: Assisted exploration of difficult transitions in humanoid motion tracking
Source: Front Neurorobot. 2026 Sep 30;20:1920711. doi: 10.3389/fnbot.2026.1920711 (PMC13661928; doi:10.3389/fnbot.2026.1920711)
Supplement: Supplementary file 1 [file Data_Sheet_1.pdf]

Supplementary Material

1 IMPLEMENTATION DETAILS

This supplement records the observation, action, domain-randomization, and reward definitions used in the Unitree G1 tracking experiments. Most of these settings are kept consistent with BeyondMimic Liao et al. (2025), and the main implementation differences are the residual-wrench action head and the residual-relaxation mechanisms introduced by RWARL. The implementation uses a 23-DoF Unitree G1 model. The torso link is used as the motion anchor for the command and termination conditions. The residual wrench, when enabled during RWARL training, is applied at the pelvis link.

2 OBSERVATION DEFINITIONS

The policy observation group concatenates all active observation terms into a single vector, which is:

$$\mathbf{o}_t^{\pi} = \left[\mathbf{q}_t^{\star},\ \dot{\mathbf{q}}_t^{\star},\ {}^{r}\mathbf{p}_{h,t}^{\star},\ {}^{r}\mathbf{R}_{h,t}^{\star},\ \mathbf{v}_t,\ \boldsymbol{\omega}_t,\ \mathbf{q}_t,\ \dot{\mathbf{q}}_t,\ \mathbf{a}_{t-1}\right], \tag{S1}$$

where $\mathbf{q}_t^{\star}$ and $\dot{\mathbf{q}}_t^{\star}$ are the reference joint position and velocity command, h denotes the anchor body, and the superscript r indicates that the reference anchor pose is expressed in the current robot-anchor frame. Orientations in the observation are represented by the first two columns of the corresponding rotation matrix, giving a six-dimensional continuous orientation representation. The joint-position term follows the joint-position observation used by the BeyondMimic tracking environment Liao et al. (2025). The previous-action term contains only the 23 joint-action dimensions for both the baseline policy and the residual-wrench policies.

Table S1. Policy observation terms in the simulation experiments. Uniform noise is additive and applied independently to each component when listed.

Term	Definition	Dimension	Noise
Command	Reference joint state $[\mathbf{q}_t^{\star}, \dot{\mathbf{q}}_t^{\star}]$	46	None
Anchor position	Anchor position ${}^{r}\mathbf{p}_{h,t}^{\star}$	3	$[-0.25, 0.25]$
Anchor orientation	Anchor orientation ${}^{r}\mathbf{R}_{h,t}^{\star}$	6	$[-0.05, 0.05]$
Base linear velocity	Base linear velocity $\mathbf{v}_t$	3	$[-0.5, 0.5]$
Base angular velocity	Base angular velocity $\boldsymbol{\omega}_t$	3	$[-0.2, 0.2]$
Joint position	Joint position $\mathbf{q}_t$	23	$[-0.01, 0.01]$
Joint velocity	Joint velocity $\dot{\mathbf{q}}_t$	23	$[-0.5, 0.5]$
Previous action	Last joint action $\mathbf{a}_{t-1}^{J}$	23	None

The critic observation group is privileged and is not the deployment observation for the joint actor. It uses the same command, anchor pose, base velocity, joint state, and previous-action terms without observation noise, and additionally includes the current tracked-body positions and orientations expressed in the robot-anchor frame. With 12 tracked bodies, these privileged body terms contribute $12 \times 3 = 36$ position dimensions and $12 \times 6 = 72$ orientation dimensions. In the residual-wrench policies, the actor policy that outputs the 23D joint action uses the policy observation, while the residual policy that outputs the 6D residual action uses the privileged observation.

For the hardware-deployed no-state-estimation variant, the implementation removes the anchor-position observation and base-linear-velocity observation from the policy input. This avoids requiring observations that are not directly available on the robot at runtime.

3 ACTION DEFINITIONS

The baseline action is a 23D joint-position target command. Each raw joint action is scaled by a joint-specific action scale and interpreted as an offset from the nominal joint target used by the simulator. For the G1 model, the scale is derived from the actuator configuration using the simulated effort limit and position-control stiffness of each joint.

The RWARL residual-action environment augments this joint action with a six-dimensional residual action $\hat{\mathbf{a}}_t$,

$$\tilde{\mathbf{a}}_t = \left[\mathbf{a}_t^J,\ \hat{\mathbf{a}}_t\right] \in \mathbb{R}^{29}, \qquad \hat{\mathbf{a}}_t = \begin{bmatrix}\boldsymbol{\delta}_{p,t} \\ \boldsymbol{\delta}_{\tau,t}\end{bmatrix} \in \hat{\mathcal{A}} \subseteq \mathbb{R}^6. \tag{S2}$$

The implementation scales the raw residual-network outputs by 0.25; the resulting position and orientation offsets are denoted by $\boldsymbol{\delta}_{p,t}$ and $\boldsymbol{\delta}_{\tau,t}$, matching the notation in the main manuscript. The implementation latches the current root position $\bar{\mathbf{p}}_t$ when the action is processed. The residual force and torque applied at the pelvis are then computed as

$$\mathbf{R}_{d,t} = \mathbf{R}^{\star}_{\text{pelvis},t}\,\mathrm{Exp}\left(\boldsymbol{\delta}_{\tau,t}\right), \tag{S3}$$

$$\mathbf{f}_{r,t} = \rho K_p^f (\bar{\mathbf{p}}_t + \boldsymbol{\delta}_{p,t} - \mathbf{p}_t) - K_d^f \mathbf{v}_t, \tag{S4}$$

$$\boldsymbol{\tau}_{r,t} = \rho K_p^\tau \mathbf{R}_t\,\mathrm{Log}\left(\mathbf{R}_t^\top \mathbf{R}_{d,t}\right) - K_d^\tau \boldsymbol{\omega}_t. \tag{S5}$$

Here $K_p^f = 1000$, $K_p^\tau = 100$, and ρ is the residual-assistance ratio. Stage 1 keeps the residual curriculum disabled with $\rho = 1$. Stage 2 enables the residual-scale curriculum, initializes $\rho = 1$, clips it to $[10^{-4}, 1]$, and reduces it as the per-segment failure-rate criterion is satisfied. The action-rate penalty is computed only on the 23 joint-action dimensions, so the residual head is regularized by the residual-wrench terms rather than by the joint action-rate term.

4 DOMAIN RANDOMIZATION AND RESET PERTURBATIONS

Table S2 summarizes the randomized physical parameters and external perturbations. Startup randomizations are sampled when the vectorized environments are created.

Table S2: Domain randomization and perturbation settings used by the tracking task.

Randomization	Range
Static friction	$[0.3, 1.6]$
Dynamic friction	$[0.3, 1.2]$
Restitution	$[0.0, 0.5]$
Torso center of mass (x)	$[-0.025, 0.025]$ m
Torso center of mass (y, z)	$[-0.05, 0.05]$ m
Random push interval	$[1.0, 3.0]$ s

At reset, the command manager initializes the robot from a sampled motion frame and then perturbs the corresponding reference state. The active reset perturbations are listed in Table S3. Joint positions are clipped to the soft joint limits after perturbation.

Table S3: Reset perturbations applied around the sampled reference state.

Quantity	Range
Root position x, y	$[-0.05, 0.05]$ m
Root position z	$[-0.01, 0.01]$ m
Root orientation roll, pitch	$[-0.1, 0.1]$ rad
Root orientation yaw	$[-0.2, 0.2]$ rad
Root linear velocity x, y	$[-0.5, 0.5]$ m/s
Root linear velocity z	$[-0.2, 0.2]$ m/s
Root angular velocity roll, pitch	$[-0.52, 0.52]$ rad/s
Root angular velocity yaw	$[-0.78, 0.78]$ rad/s
Joint position	$[-0.1, 0.1]$ rad

5 REWARD DEFINITIONS

The total reward is a weighted sum of the terms in Table S4. Let $\phi(\mathbf{u};\sigma) = \exp(-\|\mathbf{u}\|_2^2/\sigma^2)$, and let $d_R(\mathbf{R}_1, \mathbf{R}_2)$ denote the geodesic orientation error. For body-level terms, the squared error is averaged over the 12 tracked bodies. The body-pose rewards use the command-aligned reference body pose, denoted by $\tilde{\mathbf{p}}^\star_{i,t}$ and $\tilde{\mathbf{R}}^\star_{i,t}$, which aligns the reference body pose to the current robot-anchor yaw and horizontal anchor position before comparison. For limit and contact penalties, let $N_J = 23$ denote the joint-action dimension, let $[x]_+ = \max(x, 0)$, let q_j^- and q_j^+ denote the soft joint-position limits of joint j, and let $\mathcal{B}_u$ denote the undesired-contact body set.

Table S4: Reward terms used in the tracking task. Weights marked with $\star$ are used only for our residual-wrench policy.

Term	Definition	Weight
Anchor position	$\exp(-\|\mathbf{p}^\star_{h,t} - \mathbf{p}_{h,t}\|_2^2/0.3^2)$	0.5
Anchor orientation	$\exp(-d_R(\mathbf{R}^\star_{h,t}, \mathbf{R}_{h,t})^2/0.4^2)$	0.5
Body position	$\exp\left(-\frac{1}{B}\sum_{i=1}^{B}\|\tilde{\mathbf{p}}^\star_{i,t} - \mathbf{p}_{i,t}\|_2^2/0.3^2\right)$	1.0
Body orientation	$\exp\left(-\frac{1}{B}\sum_{i=1}^{B} d_R(\tilde{\mathbf{R}}^\star_{i,t}, \mathbf{R}_{i,t})^2/0.4^2\right)$	1.0
Body linear velocity	$\exp\left(-\frac{1}{B}\sum_{i=1}^{B}\|\mathbf{v}^\star_{i,t} - \mathbf{v}_{i,t}\|_2^2/1.0^2\right)$	1.0

Table S4 (continued).

Term	Definition	Weight
Body angular velocity	$\exp\left(-\frac{1}{B}\sum_{i=1}^{B}\|\boldsymbol{\omega}^{\star}_{i,t}-\boldsymbol{\omega}_{i,t}\|_2^2/\pi^2\right)$	1.0
Joint action rate	$\sum_{j=1}^{N_J}(a^J_{j,t}-a^J_{j,t-1})^2$	-0.1
Joint limit	$\sum_{j}\left([q_j^- - q_{j,t}]_+ + [q_{j,t}-q_j^+]_+\right)$	-10.0
Undesired contact	$\sum_{b\in\mathcal{B}_u}\mathbb{I}\left[\max_{\ell}\|\mathbf{F}_{b,t-\ell}\|_2 > 1.0\right]$	-0.1
Residual force regularization	$\exp(-\|\mathbf{f}_{r,t}\|_2^2/\sigma_f)$	$0.1^{\star}$
Residual torque regularization	$\exp(-\|\boldsymbol{\tau}_{r,t}\|_2^2/\sigma_\tau)$	$0.1^{\star}$

The residual regularization scales are initialized to $\sigma_f = 2.0 \times 10^7$ and $\sigma_\tau = 2.0 \times 10^6$. Following the adaptive mechanism in Kungfubot Xie et al. (2026), during residual training, each scale is updated by an exponential moving average of the corresponding squared residual-wrench magnitude with coefficient 10^{-3} and a non-increasing update rule. If the residual action term is absent, both residual regularization terms return zero and have zero effective contribution.

REFERENCES

Liao, Q., Truong, T. E., Huang, X., Gao, Y., Tevet, G., Sreenath, K., et al. (2025). Beyondmimic: from motion tracking to versatile humanoid control via guided diffusion. doi:10.48550/arXiv.2508.08241

Xie, W., Han, J., Zheng, J., Li, H., Liu, X., Shi, J., et al. (2026). Kungfubot: physics-based humanoid whole-body control for learning highly-dynamic skills. *Advances in Neural Information Processing Systems* 38, 62406–62433
